# Effect of Using Fructooligosaccharide and Isomaltooligosaccharides in Cocoa–Hazelnut Spread as Sugar Substitute and Prebiotic on Estimated Glycemic Index and Quality Parameters

**DOI:** 10.3390/foods15132402

**Published:** 2026-07-07

**Authors:** Muge Kardes, Zeynep Hazal Tekin-Cakmak, Hatice Bekiroglu, Abdullah Baycar, Salih Karasu, Mustafa Tahsin Yilmaz, Muhammed Zahid Kasapoglu, Osman Sagdic

**Affiliations:** 1 School of Tourism and Hotel Management, Gastronomy and Culinary Arts, Siirt University, 56100 Siirt, Türkiye; 2 Chemical and Metallurgical Engineering Faculty, Food Engineering Department, Yildiz Technical University, 34210 Istanbul, Türkiye; 3 Faculty of Agriculture, Department of Food Engineering, Sirnak University, 73000 Sirnak, Türkiye; 4 Department of Industrial Engineering, Faculty of Engineering, King Abdulaziz University, Jeddah 21589, Saudi Arabia; 5 Department of Nanotechnology, Institute of Nanotechnology and Biotechnology, Istanbul University-Cerrahpasa, Avcılar, 34320 Istanbul, Türkiye

**Keywords:** cocoa–hazelnut spreads, sugar substitute, estimated glycemic index, prebiotic

## Abstract

Sucrose plays a critical role in fat-continuous confectionery systems such as cocoa–hazelnut spreads by contributing to sweetness, texture, flow behavior, particle interactions, and overall sensory quality. In this study, sucrose was replaced with fructooligosaccharide (FOS) and isomaltooligosaccharide (IMO) at replacement levels of 0, 25, 50, 75, and 100% in cocoa–hazelnut spread formulations. The effects of these substitutions on physicochemical, textural, and rheological properties, particle size distribution, color parameters, estimated glycemic index (eGI), and sensory characteristics were investigated. Sucrose replacement significantly altered the structural and rheological properties of the spread matrix depending on both the type of oligosaccharide and the level of substitution. Formulations containing FOS exhibited more pronounced reductions in firmness and sensory acceptability at higher replacement levels. In contrast, IMO-containing formulations better preserved particle dispersion, viscoelastic stability, and overall sensory quality, particularly at low to moderate substitution levels. However, complete replacement of sucrose showed an adverse effect on taste and spreadability in both oligosaccharide systems. Both oligosaccharides contributed to a reduction in the eGI values. The eGI decreased from 50.71 to 39.90 in FOS-containing formulations and from 51.39 to 41.94 in IMO-containing formulations. These findings suggest that FOS is more effective in lowering the eGI, whereas IMO demonstrates greater potential for maintaining the technological and sensory quality of cocoa–hazelnut spreads when used as a partial sucrose replacer.

## 1. Introduction

Cocoa–hazelnut spreads are popular confectionery products consumed mainly at breakfast, but also during various eating occasions throughout the day [1]. These products, typically formulated with cocoa powder, hazelnut paste, milk powder, vegetable fat, sugar, and emulsifiers, occupy an important position in the market owing to their characteristic cocoa–hazelnut flavor, spreadable texture, creamy mouthfeel, and high consumer acceptance. However, their high sugar and fat contents have increased interest in reformulation strategies aimed at reducing sucrose content while maintaining desirable sensory, textural, and stability-related properties [2]. Therefore, reformulation strategies aimed at reducing sugar content while maintaining sensory quality, textural properties, and product stability have gained increasing importance in the confectionery industry.

Cocoa–hazelnut spreads are structurally complex semi-solid systems in which sugar, cocoa, milk powder, and hazelnut-derived particles are dispersed within a continuous fat phase [3]. In such systems, sucrose is not only responsible for sweetness but also acts as a key structural component that influences particle volume fraction, flow behavior, viscosity, spreadability, texture, mouthfeel, and physical stability [4,5]. Consequently, reducing or replacing sucrose may affect not only the nutritional profile of the product but also its rheological behavior and physical stability. In particular, the effects of sugar replacement on particulate network organization, viscoelastic properties, and sensory perception in fat-continuous cocoa–hazelnut spread matrices require detailed investigation [1].

In this context, the partial or complete replacement of sucrose with functional oligosaccharides represents a promising approach for improving the nutritional profile of cocoa–hazelnut spreads. Fructooligosaccharides (FOS) are short-chain fructans composed of β-(2→1)-linked fructosyl units, generally with a terminal glucose moiety, and typically exhibit a degree of polymerization ranging from 2 to 10 [6]. Due to their molecular structure, FOS are resistant to digestion by human gastrointestinal enzymes. FOS are low-calorie carbohydrates that exhibit relatively high stability under acidic conditions and thermal resistance during food processing [7]. Naturally present in several edible plants, FOS reach the colon largely intact, where they are selectively fermented by beneficial intestinal bacteria, particularly bifidobacteria, resulting in the production of short-chain fatty acids and contributing to gut health [8,9]. Owing to these properties, FOS are regarded as prebiotic ingredients and have been associated with potential benefits such as improved intestinal function, enhanced mineral absorption, modulation of lipid metabolism, and support of immune-related responses [6,10,11].

In addition to FOS, isomaltooligosaccharides (IMO) have attracted attention as alternative carbohydrate-based sweeteners and functional ingredients in reduced-sugar food systems [12,13]. IMO are mainly composed of α-(1→6)-linked glucose oligomers and are valued for their mild sweetness, technological functionality, and potential prebiotic effects. Their incorporation into cocoa–hazelnut spread formulations may contribute not only to sugar reduction but also to modifications in viscosity, particle interactions, mouthfeel, spreadability, and structural stability [14,15]. However, because FOS and IMO differ in molecular structure, sweetness profile, hygroscopicity, solubility, and interactions with dispersed particles in fat-continuous matrices, their effects on cocoa–hazelnut spread systems cannot be assumed to be identical.

Beyond their functional properties, FOS and IMO may offer advantages over sucrose in terms of reduced or slower glycemic response, making them relevant ingredients for the development of reduced-sugar products. Considering the increasing prevalence of hyperinsulinemia, insulin resistance, and diet-related metabolic disorders, replacing sucrose with lower-glycemic or slowly digestible carbohydrate alternatives has become an important strategy for developing healthier confectionery products [16,17]. Therefore, the use of oligosaccharides in cocoa–hazelnut spread formulations may provide a dual benefit by reducing sucrose content while enhancing the functional and nutritional value of the final product.

Although reduced-sugar chocolate and cocoa–hazelnut-based spread products have received growing attention, there is still limited understanding of how FOS and IMO substitution affects the physicochemical, rheological, textural, structural, sensory, and glycemic properties of these complex fat-continuous systems. Previous studies have mainly focused on conventional chocolate formulations or evaluated the effects of individual sugar replacers, such as inulin, stevia, or other carbohydrate-based alternatives, on selected quality parameters [1,4,14]. In particular, the relationships among viscoelastic behavior, flow properties, particle size distribution, textural attributes, color, sensory perception, and estimated glycemic index in cocoa–hazelnut spread matrices with progressive sucrose replacement by FOS and IMO remain insufficiently understood.

The present study aimed to investigate the effects of progressive sucrose replacement with FOS and IMO at replacement levels of 0, 25, 50, 75, and 100% on the physicochemical, textural, flow, viscoelastic, particle size, color, sensory, and estimated glycemic properties of cocoa–hazelnut spreads containing hazelnut paste. This study further aimed to provide insight into the role of different oligosaccharides in modulating particulate network organization and structural stability within a complex fat-continuous cocoa–hazelnut spread matrix. The results are expected to support the development of functional reduced-sugar cocoa–hazelnut spreads with improved nutritional quality while preserving desirable technological and sensory characteristics.

## 2. Materials and Methods

### 2.1. Materials

Cocoa–hazelnut spread samples were prepared using powdered sugar (SMS Kopuz, Istanbul, Türkiye), skimmed milk powder (Enka, Konya, Türkiye), whey powder (Enka, Konya, Türkiye), cocoa powder (Tito, Izmir, Türkiye), anhydrous vegetable fat (Orkide-41, Orkide, Izmir, Türkiye), hazelnut paste (Segmen, Giresun, Türkiye), fructooligosaccharide (FOS) (Azelis Kimya, Istanbul, Türkiye), isomaltooligosaccharide (IMO) ( VitaFiber™, Azelis Kimya, Istanbul, Türkiye), soy lecithin (Brenntag Kimya, Istanbul, Türkiye), polyglycerol polyricinoleate (PGPR) (Palsgaard, Zierikzee, The Netherlands), hazelnut flavor (Etol Frutarom, Kocaeli, Türkiye), and ethyl vanillin (Ekin Kimya, Istanbul, Türkiye). Pepsin (Sigma-Aldrich, St. Louis, MO, USA, P7000) and pancreatin (Sigma-Aldrich, St. Louis, MO, USA, P7545) were used as enzymes in the in vitro digestion procedure. Amyloglucosidase (14.45 U, 3300 U/mL) and the glucose oxidase–peroxidase (GOPOD) reagent kit were obtained from Megazyme International (Wicklow, Ireland). All chemicals and reagents used in the analyses were of analytical grade.

The fructooligosaccharide used in this study was GOFOS P95/GL, a short-chain fructooligosaccharide product with a minimum total FOS purity of 95% on a dry substance basis and a dry substance content of 95%. According to the supplier specification, GOFOS P95/GL consists mainly of GF2, GF3, and GF4 fractions, with GF2 ranging from 30 to 44%, GF3 from 45 to 57%, and GF4 from 5 to 16%.

The isomaltooligosaccharide used in this study was VitaFiber™ IMO (Edmonton, AB, Canada) powder produced from tapioca starch. It contains more than 99.5% total carbohydrates on a dry solids basis. The IMO fraction with DP ≥ 3 accounts for at least 60%, while isomaltose is ≤30% and glucose plus maltose are ≤10%. The loss on drying is specified as ≤4%. VitaFiber™ IMO is described as an IMO mixture mainly composed of glucose oligomers with a degree of polymerization ranging from 2 to 9, linked predominantly by digestion-resistant α-(1,6) glycosidic bonds.

### 2.2. Sample Preparation

Cocoa–hazelnut spread samples were produced using a pilot-scale ball mill system at the R&D center of Tayaş Gıda (Gebze, Kocaeli, Türkiye). Each formulation consisted of vegetable fat (31.00 g/100 g), skimmed milk powder (11.00 g/100 g), whey powder (12.37 g/100 g), cocoa powder (4.00 g/100 g), hazelnut paste (3.00 g/100 g), lecithin (0.35 g/100 g), polyglycerol polyricinoleate (PGPR) (0.10 g/100 g), vanillin (0.03 g/100 g), and hazelnut flavor (0.15 g/100 g).

Sucrose was progressively replaced with fructooligosaccharide (FOS) or isomaltooligosaccharide (IMO) at replacement levels of 0, 25, 50, 75, and 100%. The control formulation contained 38.00 g/100 g sucrose without oligosaccharide addition. In the reduced-sugar formulations, sucrose was decreased to 28.50, 19.00, 9.50, and 0.00 g/100 g, while the corresponding amount of FOS or IMO was increased to 9.50, 19.00, 28.50, and 38.00 g/100 g, respectively. Thus, the total amount of sucrose plus oligosaccharide was kept constant at 38.00 g/100 g in all formulations.

### 2.3. Physicochemical Analysis

Water activity (a_w_) was measured using a LabMaster aw instrument (Novasina, Lachen, Switzerland) as described by Goktas et al. [2]. Homogenized samples were placed in the sample chamber and analyzed at 25 °C under controlled laboratory conditions. All measurements were performed in triplicate, and the mean values were reported. Moisture content was determined according to the method of Lonchampt and Hartel [18]. Prior to analysis, cocoa–hazelnut spread samples were finely ground and homogenized. The samples were then extracted with anhydrous methanol at 60 °C for 6 h to ensure complete dissolution of the matrix. The resulting solutions were subsequently analyzed for water content using Karl Fischer titration. All measurements were performed in triplicate.

### 2.4. Texture Analysis

The hardness of cocoa–hazelnut spreads was measured using a texture analyzer (Stable Micro Systems, TA-TXplus, Godalming, UK) equipped with a three-point bending probe. The force required to fracture the samples was obtained from the force–displacement curve. A trigger force of 0.05 N was applied, and hardness values (N) were expressed as the mean of five replicates [19].

### 2.5. Rheological Behavior

The flow behavior of melted cocoa–hazelnut spread samples was determined at 25 °C using a controlled stress/strain rheometer (Anton Paar, MCR 302, Graz, Austria) with a probe system (PP25/S) [20]. The flow behavior of mixes was identified at a shear rate from 0.1 to 100 (s^−1^). The Casson model parameters were calculated using the following equation:

(1) $$\tau^{0.5}={\tau_{0}}^{0.5}+\eta_{pl}\dot{\gamma^{n}}$$

where τ is shear stress (Pa), γ is shear rate (s^−1^), τ_0_ is the yield stress (Pa), and η_pl_ is plastic viscosity (Pa·s).

The linear viscoelastic region (LVR) was first established by performing strain sweep tests between 0.1 and 100% strain with a parallel-plate system. Frequency sweep measurements were then carried out at a fixed strain within the LVR across 0.1–10 Hz, equivalent to approximately 0.63–62.8 rad/s. The resulting storage modulus (G′) and loss modulus (G″) values were used to assess the dynamic viscoelastic behavior of the samples. Power law parameters describing the frequency dependence of G′ and G″ were obtained by non-linear regression analysis.

(2) $$G^{\prime }=K^{\prime }\left(\omega \right)n^{\prime }$$

(3) $$G^{\prime \prime }=K^{\prime \prime }\left(\omega \right)n^{\prime \prime }$$

The thermal rheological behavior of the cocoa–hazelnut spread samples was assessed by temperature sweep analysis. The variation in storage modulus (G′) was recorded between 20 and 80 °C. During the measurement, the temperature was increased in 5 °C increments, and the samples were equilibrated for 1 min at each temperature step [21].

### 2.6. Particle Size

Particle size was determined using a micrometer (Mitutoyo Manufacturing Co., Ltd., Kawasaki, Japan; 0.001 mm precision). Measurements were performed in triplicate for each sample group [19].

### 2.7. Color and Color Stability Analysis

Color parameters (L*, a*, b*) of the samples were measured using a colorimeter (Chroma Meter CR-400, Konica Minolta, Tokyo, Japan). Chroma (C*), hue angle (*h°*), and whiteness index (*WI*) were calculated using the following equations [22]:

(4) $$C^{*}\;=\;\sqrt{{a^{*}}^{2}+\;{b^{*}}^{2}}$$

(5) $$h{}^{\circ}=\arctan\;(b^{*}/a^{*})\;$$

(6) $$WI=100-{\left[{\left(100-L\right)}^{2}+a^{2}+b^{2}\right]}^{1/2}$$

*ΔE* = [(ΔL*)2 + (Δa*)2 + (Δb*)2]1/2 (7)

### 2.8. Estimated Glycemic Index Analysis

The in vitro estimated glycemic index (eGI) of the samples was determined according to a modified Englyst et al. [23] method. Briefly, 100 mg of the sample was mixed with 2 mL of 0.05 M HCl containing pepsin (5 mg/mL) and incubated at 37 °C for 30 min with shaking. After pH adjustment with 4 mL sodium acetate buffer (0.5 M, pH 5.2), 1 mL of enzyme solution (0.104 g pancreatin and 14.45 U amyloglucosidase) was added. Aliquots were withdrawn at 0 and 90 min, mixed with ethanol, centrifuged, and glucose content was determined using the glucose oxidase–peroxidase (GOPOD, Megazyme International Ireland Ltd., Wicklow, Ireland) reagent by measuring a UV–vis spectrophotometer (Shimadzu, UV–vis 1800, Kyoto, Japan). In this assay, glucose is oxidized by glucose oxidase to produce hydrogen peroxide, which reacts in the presence of peroxidase to form a colored compound.

The hydrolysis index (HI) was calculated as the ratio of the area under the hydrolysis curve of the sample to that of white bread [24]. The eGI values were derived from HI using the equations proposed by Goñi et al. [24].

(8) $$HI=\frac{The\;area\;beneath\;the\;digestion\;curve\;of\;sample}{The\;area\;beneath\;the\;digestion\;curve\;of\;white\;bread}$$

(9) GI =39.71 + 0.549 HI

### 2.9. Sensory Analysis

The sensory evaluation of cocoa–hazelnut spread samples was conducted according to the method described by Icyer et al. [25], with minor modifications. The samples were mixed homogeneously before analysis and presented to panelists in coded containers at room temperature. Water and unsalted crackers were provided for palate cleansing between samples to minimize carryover effects. The panelists evaluated the samples in terms of appearance, brightness, color, taste, smell, and spreadability using a 5-point hedonic scale, where 1 represented the lowest acceptability and 5 represented the highest acceptability.

### 2.10. Statistical Analysis

Results were expressed as mean ± standard deviation. One-way ANOVA was performed separately for FOS- and IMO-containing formulation series to evaluate the effect of replacement level on the measured parameters. Statistical analyses were performed using IBM SPSS Statistics 27 software.

## 3. Result and Discussion

### 3.1. Physicochemical, Textural and Flow Behavior Properties

The physicochemical, textural, and flow behavior properties of fructooligosaccharide (FOS)- and isomaltooligosaccharide (IMO)-substituted cocoa–hazelnut spreads are presented in Table 1. These parameters are critical for product stability, shelf life, and sensory perception. Afoakwa [26] emphasized the strong interactions between product composition and moisture, highlighting the necessity of evaluating these criteria. Excessive moisture may reduce hardness, increase viscosity, and impair melting behavior [27], while also contributing to undesirable sensory attributes [28].

In this study, FOS-substituted and IMO-substituted samples exhibited moisture values between 1.24 and 2.01 g/100 g and 1.71 and 2.21 g/100 g, respectively (Table 1). The highest value was comparable to previous reports for control formulations [29]. Although substitution slightly increased moisture content, the differences were not statistically significant (*p* > 0.05). In the FOS series, moisture values increased gradually with increasing substitution level, whereas a less pronounced but similar trend was observed in the IMO series. The moisture values obtained in this study were generally consistent with those reported for spread-type chocolate formulations (1.46–2.00 g/100 g) by Baycar et al. [19], although slightly lower and higher values were observed depending on formulation composition. The slight increase in moisture content may be related to the different physicochemical characteristics of oligosaccharides compared with sucrose, which can influence water distribution and mass transfer behavior in food systems [7].

Water activity values of all formulations ranged between 0.21 and 0.24, indicating that oligosaccharide incorporation did not substantially alter water availability within the systems. Similar findings have been reported in reduced-sugar chocolate formulations where alternative carbohydrates mainly influenced structural organization rather than water activity [14]. In general, minor variations were observed between the FOS and IMO series, with slightly lower values at intermediate substitution levels compared with the control formulations. Moisture content slightly increased with increasing oligosaccharide concentration, particularly at higher substitution levels. This behavior may be attributed to the hygroscopic nature of oligosaccharides, which can retain higher amounts of water within the matrix. Overall, the combined results suggest that water binding and distribution were more affected by formulation structure than by total water activity. These results remained below the critical threshold of 0.40, which is considered optimal for confectionery stability [29,30,31].

Particle size distribution is a critical determinant of mouthfeel, smoothness, spreadability, and rheological behavior in chocolate-based suspensions. Large particles, particularly those above approximately 35 µm, may impart a sandy or coarse mouthfeel, whereas smaller particles generally contribute to smoother texture and improved sensory perception, although they may also influence viscosity through increased surface area and particle–particle interactions [32,33,34]. In the present study, sucrose replacement affected particle size values differently depending on the type of oligosaccharide used. In FOS-containing formulations, particle size decreased from 24.66 µm in FC000 to 18.33 and 16.66 µm in FC025 and FC050, respectively, followed by a slight increase to 23.66 and 21.00 µm in FC075 and FC100. This non-linear variation suggests that partial FOS incorporation may initially improve particle dispersion, whereas higher replacement levels may lead to partial reorganization of the dispersed solid phase. In contrast, IMO-containing formulations showed a more consistent reduction in particle size values, decreasing from 28.00 µm in IC000 to 14.33 µm in IC100. The smaller particle size observed in IMO formulations may indicate improved dispersion, particle packing efficiency, and more homogeneous particulate organization within the fat-continuous matrix. Since particle size distribution strongly influences rheological behavior, spreadability, and sensory perception in chocolate-based systems, the lower particle size values obtained in IMO-containing samples may partly explain their comparatively better technological performance at low and moderate substitution levels [4,35]. The presence of milk proteins and the combined emulsifier system composed of lecithin and PGPR may also have contributed to these differences by modifying particle surface interactions and lubrication behavior. These findings indicate that FOS and IMO modified particulate organization through different mechanisms, with IMO producing a more pronounced reduction in particle size and potentially more homogeneous dispersion within the cocoa–hazelnut spread matrix.

Texture analysis demonstrated that sucrose replacement significantly affected the structural resistance of cocoa–hazelnut spread formulations. In FOS-containing samples, firmness was maintained at 25 and 50% replacement levels, with values of 251.91 and 232.65 g, respectively, compared with 228.32 g in the control formulation. However, a marked decrease was observed at higher replacement levels, reaching 160.13 g in FC075 and 115.43 g in FC100. The decrease became particularly evident at high replacement levels, especially in FC075 and FC100, indicating that excessive FOS incorporation weakened the mechanical resistance of the spread matrix. These findings indicate weakening of the original sucrose-based particulate network. Similar reductions in structural strength and hardness after sucrose replacement with fructooligosaccharides and related carbohydrates have been reported in chocolate systems due to weaker particle–particle interactions and altered solid phase organization [14]. In contrast, IMO-containing formulations exhibited comparatively higher structural stability. Although firmness values decreased with increasing IMO concentration, the reductions were less pronounced than those observed in FOS-containing formulations. IC050 and IC075 maintained relatively high firmness and work of shear values compared to equivalent FOS samples, suggesting that IMO may better preserve the technological functionality of sucrose within the cocoa–hazelnut spread matrix.

**Figure 1:**
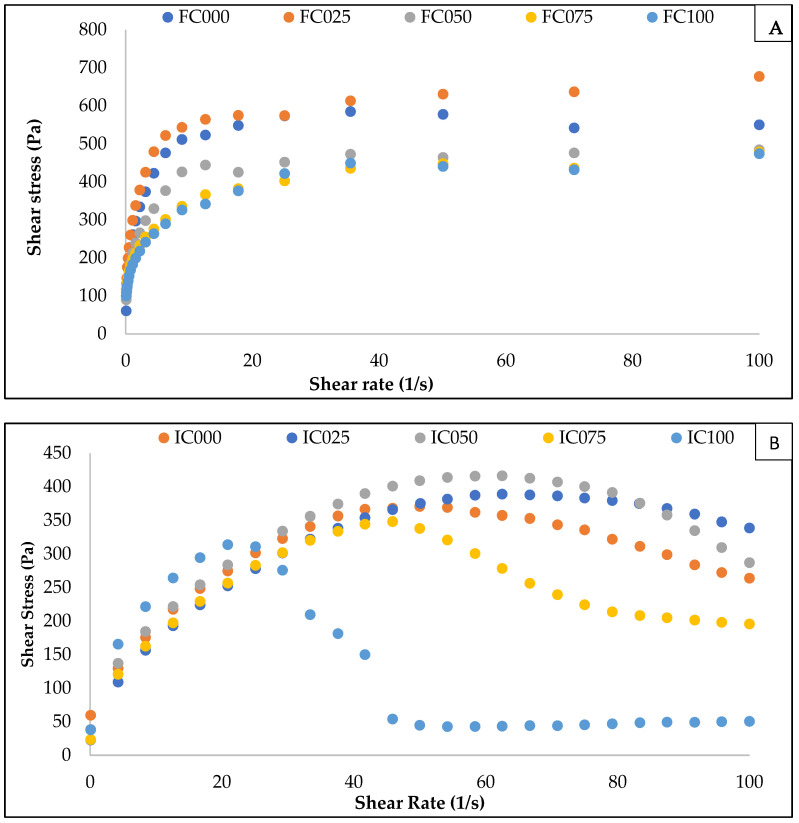
Flow behavior of fructooligosaccharide-substituted (**A**) and isomaltooligosaccharide-substituted (**B**) cocoa–hazelnut spreads. FC000–FC100: Cocoa–hazelnut spreads containing 0–100% fructooligosaccharide (FOS) as sucrose replacer. IC000–IC100: Cocoa–hazelnut spreads containing 0–100% isomaltooligosaccharide (IMO) as sucrose replacer.

Rheological behavior is a critical quality parameter in cocoa–hazelnut spread systems because it determines flowability, spreadability, mouthfeel, and processing performance. In fat-continuous chocolate-based systems, flow properties are strongly governed by the interactions among dispersed solid particles, fat phase, emulsifiers, and bulk sweeteners [4,33]. The flow behavior parameters of FOS- and IMO-substituted cocoa–hazelnut spreads are presented in Table 1 and Figure 1. The Casson model adequately described the flow behavior of the formulations, with R^2^ values ranging from 0.879 to 0.976. These values indicate that the model reasonably represented the flow characteristics of the cocoa–hazelnut spread systems, although the fitting performance varied depending on oligosaccharide type and replacement level.

In FOS-containing formulations, the Casson yield stress decreased from 78.10 Pa in FC000 to 41.19 Pa in FC100, indicating that increasing FOS replacement reduced the stress required to initiate flow. This decrease suggests weakening of the initial particulate network and lower resistance to deformation at high FOS levels. However, the response was not strictly linear, as FC050 showed a higher yield stress than FC025 and FC075. This non-linear behavior indicates that FOS incorporation modified the internal organization of the fat-continuous matrix through changes in particle packing, interparticle interactions, and lubrication behavior rather than producing a simple concentration-dependent effect. Similar behavior has been reported in chocolate systems, where bulk sweeteners altered yield stress and plastic viscosity by modifying solid volume fraction, particle size distribution, and interparticle contacts [4].

The Casson plastic viscosity of FOS-containing samples increased from 2.64 Pa·s in FC000 to 4.26 Pa·s in FC100. This increase suggests that although high FOS replacement reduced the initial yield stress, it increased viscosity-related resistance during continued flow. Such behavior may be associated with changes in the continuous phase, altered interactions among dispersed solids, or the formation of an oligosaccharide-rich matrix that restricts flow after deformation begins. This interpretation is consistent with the view that carbohydrate-based replacers act not only as sweetening agents but also as structure-modifying components in chocolate matrices [14,35].

IMO-containing formulations showed a different flow behavior pattern. The Casson yield stress decreased from 79.75 Pa in IC000 to approximately 50 Pa in substituted samples, indicating that IMO replacement generally reduced the initial flow resistance compared with the control. The Casson plastic viscosity varied between 2.55 and 4.49 Pa·s, with the highest value observed in IC075. Compared with FOS-containing samples, IMO formulations maintained relatively moderate plastic viscosity values across the substitution range. This behavior may be associated with the smaller particle size values observed in IMO-containing formulations, which could have improved particle dispersion and reduced frictional resistance within the fat-continuous matrix. Van de Walle et al. [35] similarly emphasized that the physicochemical properties of bulking agents are closely related to chocolate quality attributes, including rheological behavior and sensory-related properties.

The differences between FOS and IMO formulations demonstrate that the rheological behavior of reduced-sugar cocoa–hazelnut spreads depends strongly on the type of oligosaccharide used. In chocolate and fat-continuous spread systems, sucrose particles contribute not only to sweetness but also to particle packing, lubrication, yield behavior, and viscosity [4,36]. Therefore, replacing sucrose with oligosaccharides can alter the structural organization of the dispersed phase. The present results indicate that FOS and IMO modified the flow behavior of cocoa–hazelnut spreads through different mechanisms. FOS replacement, particularly at high levels, lowered yield stress but increased plastic viscosity, suggesting easier flow initiation but greater resistance during continued shearing. In contrast, IMO replacement generally reduced yield stress while maintaining moderate plastic viscosity values, indicating a more balanced flow profile within the spread matrix. This supports the broader interpretation that IMO may better preserve technological functionality, whereas FOS may cause greater changes in flow resistance depending on replacement level.

### 3.2. Viscoelastic Properties

**Figure 2:**
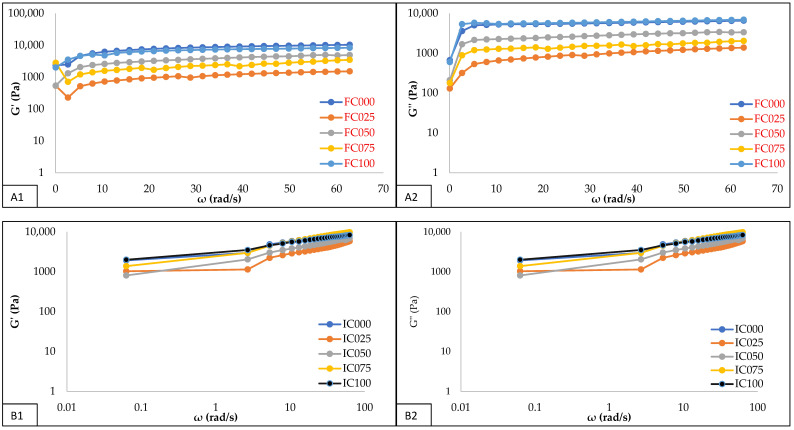
Dynamic viscoelastic behavior of fructooligosaccharide-substituted (**A1**,**A2**) and isomaltooligosaccharide-substituted (**B1**,**B2**) cocoa–hazelnut spreads. FC000–FC100: Cocoa–hazelnut spreads containing 0–100% fructooligosaccharide (FOS) as sucrose replacer. IC000–IC100: Cocoa–hazelnut spreads containing 0–100% isomaltooligosaccharide (IMO) as sucrose replacer.

The viscoelastic properties of FOS- and IMO-substituted cocoa–hazelnut spreads are presented in Table 2 and Figure 2. All formulations exhibited viscoelastic behavior typical of concentrated fat-continuous cocoa–hazelnut spreads. However, the magnitude of the dynamic rheological parameters varied depending on both oligosaccharide type and replacement level, indicating that sucrose substitution modified the internal particulate network of the spread matrix. The frequency-dependent changes in G′ and G″ further showed that the samples exhibited both elastic and viscous contributions, with the relative magnitudes of these moduli varying according to oligosaccharide type and replacement level. This indicates that sucrose replacement altered the balance between solid-like and liquid-like behavior within the cocoa–hazelnut spread matrix.

Among FOS-containing formulations, FC050 exhibited the most pronounced decrease in viscoelastic strength. K′ decreased from 2955.35 in the control formulation to 1034.26, while K″ decreased from 3147.33 to 1212.13. Simultaneously, the n′ value increased to 0.378, indicating stronger frequency dependence and weaker structural organization. Increased frequency dependence is generally associated with a less interconnected and less stable viscoelastic network. These findings suggest that 50% FOS replacement disrupted the original sucrose-stabilized particulate structure within the fat-continuous matrix.

Similar weakening of rheological structure after sucrose replacement has been reported in reduced-sugar chocolate systems. Lim et al. [14] showed that replacing sucrose with inulin, FOS, trehalose, or maltodextrin altered the physicochemical and sensory properties of dark compound chocolate, indicating that carbohydrate-based replacers do not behave merely as sweeteners but also as structure-modifying agents. In the present study, the decrease in K′ and K″ at 50% FOS replacement is consistent with this interpretation and may be related to changes in particle packing, interparticle interactions, and lubrication behavior within the fat-continuous phase. Sokmen and Gunes [4] also demonstrated that chocolate rheology is strongly governed by solid volume fraction and particle size distribution, with different bulk sweeteners producing distinct effects on plastic viscosity and yield stress. Therefore, the reduction in viscoelastic strength observed in FC050 may reflect disruption of the original sucrose-based particulate arrangement rather than a simple dilution effect.

At higher FOS replacement levels, the viscoelastic parameters partially recovered. FC100 exhibited K′ and K″ values higher than those of the control sample, while n′ and n″ values decreased compared with FC050. Lower frequency dependence indicates the formation of a more structured network with reduced sensitivity to frequency changes. This behavior suggests that high FOS concentrations may promote the development of a reorganized oligosaccharide-rich particulate network within the cocoa–hazelnut spread matrix. However, this increased viscoelastic strength should not be interpreted alone as improved product quality, since high FOS replacement was also associated with lower firmness and reduced sensory scores. A similar discrepancy between instrumental structuring and sensory quality has been reported in cocoa–hazelnut spreads formulated with inulin–stevia as sugar replacers, where complete or excessive sugar replacement could reduce spreadability despite maintaining certain textural or structural attributes [1]. This supports the present finding that a stronger viscoelastic network does not necessarily correspond to better consumer-oriented quality in spreadable cocoa systems.

IMO-containing formulations exhibited comparatively more stable viscoelastic behavior during sucrose replacement. The reductions in K′ and K″ values at intermediate replacement levels were less pronounced than those observed in FOS-containing samples. Moreover, IC075 and IC100 maintained relatively high viscoelastic strength combined with lower frequency dependence values, indicating improved structural continuity and network stability. This behavior is consistent with the particle size results, where IMO-containing formulations showed a more pronounced reduction in particle size values, suggesting improved dispersion and more homogeneous particulate organization. Van de Walle et al. [35] reported that the physicochemical properties of bulking agents strongly influence chocolate quality attributes, supporting the interpretation that the different molecular structures and interaction behaviors of FOS and IMO may account for their distinct effects on network formation.

The relatively stable rheological behavior observed in IMO-containing systems may be associated with improved particle packing and dispersion within the fat-continuous matrix [36]. The presence of milk proteins, whey powder, and the combined emulsifier system composed of lecithin and PGPR may also have contributed to these differences by modifying particle surface interactions, interparticle friction, and lubrication behavior. These findings indicate that FOS and IMO modified the viscoelastic properties of cocoa–hazelnut spreads through different structural mechanisms. FOS caused a more pronounced disruption at intermediate replacement levels, followed by structural recovery at full replacement, whereas IMO better preserved network continuity across the substitution range.

### 3.3. Thermal Rheological Behavior

**Figure 3:**
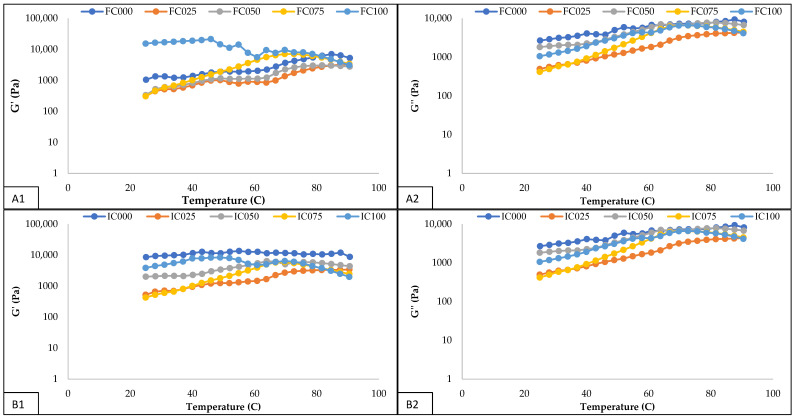
Temperature sweep test of fructooligosaccharide-substituted (**A1**,**A2**) and isomaltooligosaccharide-substituted (**B1**,**B2**) cocoa–hazelnut spreads. FC000–FC100: Cocoa–hazelnut spreads containing 0–100% fructooligosaccharide (FOS) as sucrose replacer. IC000–IC100: Cocoa–hazelnut spreads containing 0–100% isomaltooligosaccharide (IMO) as sucrose replacer.

The temperature-dependent rheological behavior of FOS- and IMO-substituted cocoa–hazelnut spreads is shown in Figure 3. Temperature sweep analysis provides insight into the structural stability of fat-continuous spread systems during heating, where changes in fat phase mobility, particle interactions, and network organization may alter storage modulus and elastic network strength [37,38,39]. In the present study, the storage modulus (G′) of the samples showed different temperature-dependent patterns depending on the oligosaccharide type and replacement level, indicating that sucrose substitution modified the thermal stability of the cocoa–hazelnut spread matrix.

Formulations containing low levels of FOS (FC025 and FC050) exhibited temperature dependency comparable to that of the sucrose-containing control sample. In these samples, the storage modulus (G′) increased with rising temperature, indicating that the elastic character of the system was preserved and the structural network was maintained. This behavior suggested that partial substitution of sucrose with FOS, up to 50%, did not markedly disrupt the intermolecular interactions responsible for the viscoelastic stability of the cocoa–hazelnut spread matrix.

In contrast, formulations containing high levels of FOS (FC075 and FC100) showed different temperature dependency characteristics. Although the samples FC075 and FC100 initially exhibited viscoelastic characteristics similar to the sugar-containing control sample and samples containing a low level of FOS (FC025 and FC050), a dramatic decline in G′ values of FC075 and FC100 was observed beyond 40 °C. The dramatic decrease in G′ values indicates a significant weakening of the structural network and a loss of resistance against thermal deformation. This result suggests that excessive replacement of sucrose with FOS adversely affected the integrity of the samples. These results demonstrate that the amount of FOS in the formulation of spreads significantly affected the thermal stability of cocoa–hazelnut spreads. The moderate levels of FOS substitution maintained the structural integrity of the system under thermal stress, but excessive concentrations of FOS disrupted network stability and weakened the sample matrix.

FC100 exhibited markedly high G′ values at the beginning of heating, suggesting the formation of a highly structured FOS-rich matrix. However, the subsequent decline in G′ at higher temperatures indicates that this structure was not fully resistant to thermal disruption. This result is consistent with the sensory and texture findings, where full FOS replacement was associated with reduced firmness and lower sensory scores despite strong viscoelastic structuring.

IMO-containing formulations also exhibited concentration-dependent thermal responses. IC025 showed relatively low G′ values throughout heating, whereas IC050 and IC075 showed an increase in G′ with increasing temperature, followed by a decrease at higher temperatures. IC100 exhibited high initial G′ values, indicating stronger structuring at full replacement; however, G′ decreased substantially after the mid-temperature range, suggesting partial breakdown or relaxation of the network during heating. Compared with FOS-containing systems, IMO formulations showed more balanced thermal behavior at intermediate replacement levels, which may be related to improved particle dispersion and more homogeneous matrix organization, as supported by the particle size results.

The increase in G′ observed in several formulations during heating may be attributed to thermally induced rearrangement of dispersed particles, changes in fat crystal structure, increased particle–particle interactions, and redistribution of solids within the continuous fat phase [37,38,39]. Conversely, the decline in G′ at higher temperatures may be associated with increased fat phase mobility, weakening of particle interactions, and partial disruption of the structured network [38,39]. These findings indicate that oligosaccharide type and replacement level strongly influenced the thermal rheological stability of cocoa–hazelnut spreads. FOS, particularly at full replacement, promoted strong initial structuring but showed signs of thermal instability at elevated temperatures, whereas IMO provided comparatively more balanced structural behavior at partial replacement levels.

### 3.4. Color Properties

Color parameters (L*, a*, b*, C*, *h°*, and *WI*) are important quality attributes for cocoa–hazelnut spreads because they directly influence visual perception and consumer acceptance [40]. The color properties of FOS- and IMO-substituted cocoa–hazelnut spread formulations are presented in Table 3. In FOS-containing samples, L* values decreased from 41.99 in FC000 to 37.70 in FC100, indicating a gradual darkening of the samples with increasing FOS replacement. Similarly, the whiteness index decreased from 40.66 to 35.13, supporting the reduction in lightness. In contrast, a* values increased from 8.47 in the control formulation to approximately 11.68 at full FOS replacement, while b* values were also higher in substituted samples than in the control. These changes indicate that FOS incorporation intensified the reddish-yellow color components of the cocoa–hazelnut spread matrix. The changes observed in the color parameters of the cocoa–hazelnut spread samples can be mainly attributed to the replacement of sucrose with FOS and IMO, which may have modified the optical properties and internal organization of the fat-continuous spread matrix. Sucrose, FOS, and IMO differ in terms of particle characteristics, solubility, hygroscopicity, and their interactions with cocoa powder, milk powder, hazelnut paste, and the fat phase. These differences may influence light scattering, surface reflection, and the distribution of solid particles within the matrix. Therefore, the variations in L*, a*, b*, C*, h°, WI, and ΔE values may be associated with formulation-dependent changes in particle dispersion and matrix homogeneity. In particular, the incorporation of oligosaccharides may alter brightness and color intensity by changing the interaction between hydrophilic carbohydrate components and the surrounding fat phase. The differences between FOS- and IMO-containing formulations may also be related to their different molecular structures and physical properties, which can affect the visual appearance of the final product.

IMO-containing formulations showed more variable changes in color parameters. The L* values ranged from 27.63 to 41.71, with IC025 showing the lowest lightness and whiteness index values, whereas IC050 exhibited the highest L* value among the IMO-containing samples. This variability may be associated with differences in particle dispersion, surface reflectance, and matrix homogeneity. The a* and b* values indicated the contribution of reddish and yellowish tones typical of cocoa-containing spread systems, and their variations suggest that sucrose replacement with IMO modified the optical properties of the matrix.

The color changes observed in the present study are partly consistent with previous studies on reduced-sugar chocolate and cocoa–hazelnut spread systems. Berk et al. [1] reported that replacing sucrose with an inulin–stevia mixture in cocoa–hazelnut spread formulations affected color parameters together with particle size distribution, textural properties, and rheological behavior, indicating that optical properties are closely linked to matrix organization and ingredient distribution. This agrees with the present findings, where changes in L*, *WI*, and chromatic parameters were accompanied by differences in particle size values and rheological behavior. However, the direction and magnitude of color changes may differ depending on the type of sugar replacer, its intrinsic color, particle size, water-binding capacity, and interaction with cocoa, milk solids, and fat phase.

Lim et al. [14] also showed that sucrose replacement with inulin, FOS, trehalose, or maltodextrin altered the physicochemical and sensory properties of dark compound chocolate. Although their product matrix differed from the cocoa–hazelnut spread system used in the present study, their findings support the view that alternative carbohydrates may change color perception indirectly by modifying solid dispersion, surface characteristics, and matrix structure. In the present study, the decrease in L* and WI values in FOS-containing samples may therefore be related not only to ingredient substitution but also to changes in light scattering caused by altered particle organization within the fat-continuous phase.

Sokmen and Gunes [4] emphasized that bulk sweeteners can modify chocolate structure through changes in solid volume fraction and particle size distribution, while Van de Walle et al. [35] showed that the physicochemical properties of bulking agents are closely associated with chocolate quality attributes. These studies help explain why FOS and IMO produced different color responses in the present study despite replacing the same amount of sucrose. The more consistent reduction in particle size observed in IMO-containing formulations may have altered surface reflectance and contributed to differences in lightness and whiteness index, whereas the non-linear particle size variation in FOS-containing samples may have resulted in different light scattering behavior. These results indicate that sucrose replacement with FOS or IMO altered the visual characteristics of cocoa–hazelnut spreads, and the observed differences were not solely related to the color of the ingredients. Instead, the changes appear to reflect oligosaccharide-induced modifications in particulate organization, matrix homogeneity, and light scattering within the fat-continuous cocoa–hazelnut spread system.

### 3.5. Sensory Properties

The sensory properties of the cocoa–hazelnut spread formulations are presented in Table 4. Oligosaccharide type and replacement level influenced appearance, brightness, color, taste, smell, and spreadability scores. In FOS-containing formulations, sensory scores generally decreased as the replacement level increased, particularly for taste, smell, and spreadability. FC025 maintained scores close to the control formulation for appearance, brightness, and spreadability, whereas FC100 received the lowest scores among FOS-containing samples, especially for taste and spreadability. This decline may be associated with changes in sweetness perception, reduced structural resistance, altered matrix organization, and the sensory impact of complete sucrose replacement.

Although FC100 exhibited relatively high viscoelastic parameters, this did not translate into improved sensory quality. This finding indicates that high rheological strength alone does not necessarily correspond to better consumer-oriented quality in spreadable products. In spread systems, excessive structuring or an unfavorable balance between firmness, flowability, and lubrication may negatively affect spreadability and mouthfeel. Similar observations were reported by Berk et al. [1], who showed that sugar replacement in cocoa–hazelnut spreads may alter sensory quality despite maintaining certain structural or technological properties.

IMO-containing formulations preserved sensory characteristics more effectively at low and moderate replacement levels. IC025 and IC050 maintained appearance, brightness, color, and spreadability scores relatively close to those of the control sample. This behavior may be related to the smaller particle size distribution and comparatively stable viscoelastic behavior observed in IMO-containing systems, which may have contributed to improved matrix homogeneity and smoother mouthfeel. However, complete sucrose replacement also negatively affected the sensory quality of IMO formulations. IC100 showed low scores for taste and spreadability, indicating that excessive IMO incorporation may impair sensory perception despite its favorable effects on particle size distribution. These results demonstrate that sucrose replacement with oligosaccharides induces complex structural and sensory modifications in cocoa–hazelnut spread systems. Low to moderate replacement levels, particularly with IMO, appear more suitable for preserving sensory quality, whereas complete sucrose replacement may be technologically feasible but sensorially limiting. Therefore, optimization of the replacement level is necessary to balance reduced sugar content, structural stability, spreadability, and sensory acceptability in cocoa–hazelnut spread formulations.

### 3.6. Estimated Glycemic Index

The estimated glycemic index (eGI) and hydrolysis index (HI) values of FOS- and IMO-substituted cocoa–hazelnut spreads are presented in Table 5. Sucrose replacement with oligosaccharides reduced the eGI values in both formulation series. In FOS-containing formulations, eGI decreased from 50.71 in FC000 to 39.90 in FC100, whereas in IMO-containing formulations, GI decreased from 51.39 in IC000 to 41.94 in IC100. According to international classifications, all formulations were within the low-GI category, as eGI values below 55 are classified as low [40]. However, oligosaccharide incorporation further reduced the eGI of the cocoa–hazelnut spread samples. The lowest eGI value was obtained in FC100, followed by IC100, indicating that complete sucrose replacement was the most effective strategy for lowering eGI.

The reduction in eGI values was accompanied by a marked decrease in HI, reflecting lower enzymatic hydrolysis during in vitro digestion as described by the modified Englyst method [23]. In FOS-containing formulations, HI decreased sharply from 20.05 in FC000 to 0.35 in FC100. In IMO-containing formulations, HI decreased from 21.28 in IC000 to 4.53 in IC100. This difference suggests that FOS was more effective than IMO in reducing carbohydrate hydrolysis and estimated glycemic index. The stronger GI-lowering effect of FOS may be associated with its resistance to digestion by human gastrointestinal enzymes and its lower contribution to glucose release during in vitro hydrolysis. IMO also reduced values, but its effect was comparatively less pronounced, possibly due to differences in glycosidic linkage structure and enzymatic susceptibility.

The present results are consistent with previous studies reporting that the incorporation of FOS or other low-digestible carbohydrates can reduce starch or carbohydrate hydrolysis and lower eGI values in different food matrices. Ishwarya and Prabhasankar [41] reported that FOS incorporation reduced estimated glycemic response-related parameters in biscuit formulations, while Megala and Hymavathi [42] observed similar glycemic-lowering effects in fruit-based products. Shanthamma et al. [43] also reported that the use of alternative sugar replacers in chocolate systems contributed to lower eGI values compared with conventional sucrose-containing formulations. These findings support the present results, where replacing sucrose with FOS and IMO reduced both eGI and HI values in cocoa–hazelnut spreads. However, the magnitude of eGI reduction in the present study differed between FOS and IMO formulations, indicating that the estimated glycemic index of reduced-sugar products depends not only on the amount of sucrose replaced but also on the molecular structure and digestibility of the replacer. The sharper decline in HI observed in FOS-containing samples suggests greater resistance to enzymatic hydrolysis compared with IMO. This may be attributed to the fructan-type structure of FOS, which is less susceptible to hydrolysis by human digestive enzymes. In contrast, IMO consists mainly of glucose-based oligosaccharides with α-glycosidic linkages, which may explain its comparatively higher HI and eGI values at equivalent replacement levels.

Shanthamma et al. [43] emphasized that the development of low-eGI chocolate products using natural sugar substitutes requires not only reducing digestible sugars but also maintaining acceptable technological and sensory quality. This is particularly important in cocoa–hazelnut spread systems, where sucrose contributes to sweetness, particle packing, texture, and flow behavior. In the present study, FOS provided the greatest reduction in eGI, whereas IMO better preserved structural and sensory quality at low and moderate replacement levels. Therefore, the results reveal a clear formulation trade-off: FOS appears more advantageous for lowering estimated glycemic index, while IMO may be more suitable when technological functionality and sensory acceptability are prioritized.

## 4. Conclusions

This study demonstrated that progressive sucrose replacement with fructooligosaccharide (FOS) and isomaltooligosaccharide (IMO) significantly affected the physicochemical, textural, rheological, structural, sensory, and estimated glycemic properties of cocoa–hazelnut spreads. Although both oligosaccharides enabled sugar reduction and contributed to lower eGI values, their effects on product quality were not identical. FOS was more effective in reducing the eGI, with the lowest eGI value observed in the fully substituted FOS formulation. However, high levels of FOS replacement, particularly at 75 and 100%, reduced firmness and sensory acceptability, indicating that complete sucrose replacement with FOS may negatively affect consumer-oriented quality attributes. IMO-containing formulations showed comparatively better preservation of technological and sensory quality, especially at low to moderate replacement levels. The smaller particle size values, more stable viscoelastic behavior, and relatively balanced flow properties observed in IMO-substituted samples suggest that IMO may better maintain the structural functionality of sucrose in fat-continuous cocoa–hazelnut spread systems. Nevertheless, complete IMO replacement also resulted in sensory limitations, particularly in taste and spreadability. These findings indicate that the selection of oligosaccharide type and replacement level is critical for balancing sugar reduction, eGI, rheological stability, texture, and sensory quality. Among the analyzed formulations, partial replacement levels appear more promising for the development of reduced-sugar cocoa–hazelnut spreads with acceptable technological and sensory characteristics. FOS may be preferred when estimated glycemic reduction is the primary objective, whereas IMO appears more suitable when maintaining spreadability, structural stability, and sensory acceptability is prioritized. Further studies including storage stability, oxidative stability, consumer acceptance, and in vivo glycemic response evaluation would provide additional insight into the commercial applicability of these formulations. In addition, future research may also explore the combined application of FOS and IMO in complex food matrices to investigate potential synergistic effects on technological and nutritional properties.

## Figures and Tables

**Table 1:** Physicochemical, textural, and flow behavior and particle size properties of fructooligosaccharide- and isomaltooligosaccharide-substituted cocoa–hazelnut spreads.

Sample	Physicochemical		Texture		Flow Behavior Parameters (Casson Model)			Particle Size
	Water Activity (a_w_)	Moisture (g/100 g)	Firmness (g)	Work of Shear (g·min)	*τ_0_* (Pa)	*η_pl_* (Pa·s)	R^2^	Particle Size (µm)
Fructooligosaccharide-substituted cocoa–hazelnut spreads								
FC000	0.24 ± 0.00 ^a^	1.57 ± 0.33 ^b^	228.32 ± 17.62 ^a^	149.86 ± 26.70 ^a^	78.10 ± 1.66 ^a^	2.64 ± 0.58 ^b^	0.976	24.66 ± 0.57 ^a^
FC025	0.22 ± 0.00 ^b^	1.95 ± 0.25 ^ab^	251.91 ± 15.84 ^a^	167.14 ± 16.38 ^a^	46.48 ± 4.79 ^b^	3.31 ± 0.18 ^ab^	0.945	18.33 ± 0.57 ^c^
FC050	0.21 ± 0.00 ^b^	1.78 ± 0.15 ^ab^	232.65 ± 36.76 ^a^	174.47 ± 41.80 ^a^	57.84 ± 7.42 ^b^	3.58 ± 0.61 ^ab^	0.951	16.66 ± 0.57 ^c^
FC075	0.22 ± 0.01 ^ab^	2.29 ± 0.01 ^a^	160.13 ± 24.95 ^b^	127.01 ± 3.22 ^ab^	41.71 ± 6.44 ^c^	4.02 ± 0.20 ^a^	0.949	23.66 ± 0.57 ^a^
FC100	0.21 ± 0.01 ^b^	2.25 ± 0.38 ^ab^	115.43 ± 6.05 ^b^	74.81 ± 10.38 ^b^	41.19 ± 3.18 ^c^	4.26 ± 0.20 ^a^	0.960	21.00 ± 1.00 ^b^
Isomaltooligosaccharide-substituted cocoa–hazelnut spreads								
IC000	0.23 ± 0.00 ^a^	1.71 ± 0.10 ^b^	269.64 ± 29.55 ^a^	197.92 ± 0.78 ^a^	79.75 ± 1.34 ^a^	3.28 ± 0.12 ^b^	0.964	28.00 ± 2.00 ^a^
IC025	0.22 ± 0.00 ^a^	2.03 ± 0.03 ^a^	211.65 ± 8.75 ^bc^	166.01 ± 11.68 ^ab^	50.70 ± 0.72 ^c^	2.55 ± 0.04 ^c^	0.879	21.33 ± 0.57 ^b^
IC050	0.22 ± 0.01 ^a^	2.21 ± 0.17 ^a^	212.2 ± 9.94 ^b^	169.65 ± 9.21 ^ab^	55.82 ± 1.12 ^b^	3.09 ± 0.09 ^b^	0.909	17.33 ± 1.52 ^c^
IC075	0.21 ± 0.00 ^a^	2.03 ± 0.14 ^a^	210.92 ± 5.03 ^bc^	171.08 ± 24.52 ^ab^	50.11 ± 0.17 ^c^	4.49 ± 0.10 ^a^	0.925	18.33 ± 1.52 ^bc^
IC100	0.22 ± 0.00 ^a^	1.95 ± 0.05 ^ab^	172.43 ± 2.56 ^c^	140.08 ± 5.49 ^b^	49.88 ± 0.80 ^c^	4.38 ± 0.02 ^a^	0.891	14.33 ± 1.15 ^c^

Different lowercase superscripts indicate significant differences between sample groups (*p* < 0.05). All experiments were performed in triplicate. All data are represented as means ± standard deviation. FC000–FC100: Cocoa–hazelnut spreads containing 0–100% fructooligosaccharide (FOS) as sucrose replacer. IC000–IC100: Cocoa–hazelnut spreads containing 0–100% isomaltooligosaccharide (IMO) as sucrose replacer.

**Table 2:** Viscoelastic properties of fructooligosaccharide- and isomaltooligosaccharide-substituted cocoa–hazelnut spreads.

Sample	Viscoelastic Parameters					
	K′ (Pa·s^n′^)	n′	R^2^	K″ (Pa·s^n″^)	n″	R^2^
Fructooligosaccharide-substituted cocoa–hazelnut spreads						
FC000	2955.350 ± 203.79 ^a^	0.305 ± 0.012 ^b^	0.984	3147.334 ± 126.40 ^b^	0.176 ± 0.011 ^d^	0.949
FC025	2221.452 ± 83.20 ^b^	0.307 ± 0.021 ^b^	0.988	2246.298 ± 55.47 ^c^	0.424 ± 0.071 ^a^	0.994
FC050	1034.259 ± 48.71 ^d^	0.378 ± 0.011 ^a^	0.997	1212.134 ± 20.64 ^e^	0.246 ± 0.008 ^b^	0.981
FC075	1972.128 ± 93.09 ^c^	0.109 ± 0.008 ^d^	0.994	1677.571 ± 103.71 ^d^	0.202 ± 0.002 ^c^	0.933
FC100	3072.163 ± 56.28 ^a^	0.242 ± 0.029 ^c^	0.993	3650.740 ± 140.17 ^a^	0.151 ± 0.024 ^d^	0.900
Isomaltooligosaccharide-substituted cocoa–hazelnut spreads						
IC000	2883.974 ± 25.11 ^ab^	0.292 ± 0.022 ^b^	0.993	2905.011 ± 145.31 ^b^	0.188 ± 0.010 ^b^	0.941
IC025	2224.567 ± 121.56 ^c^	0.255 ± 0.045 ^c^	0.929	2284.795 ± 86.17 ^c^	0.217 ± 0.002 ^a^	0.979
IC050	1722.317 ± 196.59 ^d^	0.332 ± 0.018 ^a^	0.993	2158.507 ± 103.07 ^c^	0.174 ± 0.007 ^c^	0.985
IC075	2721.236 ± 236.24 ^b^	0.311 ± 0.031 ^ab^	0.997	3363.166 ± 193.27 ^a^	0.194 ± 0.012 ^b^	0.961
IC100	3098.970 ± 159.30 ^a^	0.235 ± 0.015 ^c^	0.991	3557.188 ± 139.06 ^a^	0.145 ± 0.005 ^d^	0.861

Different lowercase superscripts indicate significant differences between sample groups (*p* < 0.05). All experiments were performed in triplicate. All data are represented as means ± standard deviation. FC000–FC100: Cocoa–hazelnut spreads containing 0–100% fructooligosaccharide (FOS) as sucrose replacer. IC000–IC100: Cocoa–hazelnut spreads containing 0–100% isomaltooligosaccharide (IMO) as sucrose replacer.

**Table 3:** Color properties of fructooligosaccharide- and isomaltooligosaccharide-substituted cocoa–hazelnut spreads.

Sample	CIE-Lab					
	L*	a*	b*	C*	*h°*	*WI*
Fructooligosaccharide-substituted cocoa–hazelnut spreads						
FC000	41.99 ± 0.15 ^a^	8.47 ± 0.04 ^c^	9.22 ± 0.25 ^d^	12.52 ± 0.20 ^d^	47.42 ± 0.67 ^c^	40.66 ± 0.19 ^a^
FC025	39.38 ± 0.28 ^b^	11.49 ± 0.14 ^a^	15.06 ± 0.22 ^a^	18.94 ± 0.22 ^c^	52.66 ± 0.75 ^a^	36.49 ± 0.29 ^b^
FC050	39.09 ± 0.15 ^b^	11.12 ± 0.15 ^b^	14.15 ± 0.30 ^bc^	18.00 ± 0.13 ^b^	51.82 ± 0.99 ^a^	36.49 ± 0.18 ^b^
FC075	38.35 ± 0.05 ^c^	11.67 ± 0.00 ^a^	14.46 ± 0.03 ^b^	18.58 ± 0.02 ^a^	51.10 ± 0.06 ^cb^	35.61 ± 0.05 ^c^
FC100	37.70 ± 0.19 ^d^	11.68 ± 0.10 ^a^	13.78 ± 0.20 ^c^	18.06 ± 0.12 ^b^	49.69 ± 0.60 ^b^	35.13 ± 0.16 ^c^
Isomaltooligosaccharide-substituted cocoa–hazelnut spreads						
IC000	36.52 ± 2.93 ^ab^	8.67 ± 0.90 ^e^	14.44 ± 1.84 ^b^	16.84 ± 2.04 ^a^	58.95 ± 0.63 ^b^	34.28 ± 2.38 ^ab^
IC025	27.63 ± 6.30 ^b^	9.69 ± 0.84 ^b^	17.44 ± 0.79 ^a^	19.95 ± 1.09 ^a^	60.98 ± 1.07 ^a^	24.89 ± 5.81 ^b^
IC050	41.71 ± 1.96 ^a^	9.30 ± 0.39 ^d^	16.08 ± 0.79 ^ab^	18.58 ± 0.88 ^a^	59.94 ± 0.24 ^ab^	38.81 ± 1.62 ^a^
IC075	37.95 ± 4.39 ^ab^	9.47 ± 0.38 ^a^	16.62 ± 0.48 ^ab^	19.12 ± 0.60 ^a^	60.33 ± 0.41 ^ab^	35.05 ± 4.04 ^a^
IC100	37.28 ± 2.06 ^ab^	9.13 ± 0.32 ^c^	15.51 ± 1.02 ^ab^	18.00 ± 1.03 ^a^	59.48 ± 0.87 ^ab^	34.74 ± 2.18 ^a^

Different lowercase superscripts indicate significant differences between sample groups (*p* < 0.05). All experiments were performed in triplicate. All data are represented as means ± standard deviation. FC000–FC100: Cocoa–hazelnut spreads containing 0–100% fructooligosaccharide (FOS) as sucrose replacer. IC000–IC100: Cocoa–hazelnut spreads containing 0–100% isomaltooligosaccharide (IMO) as sucrose replacer.

**Table 4:** Sensory properties of fructooligosaccharide- and isomaltooligosaccharide-substituted cocoa–hazelnut spreads.

Sample	Appearance	Brightness	Color	Taste	Smell	Spreadability
Fructooligosaccharide-substituted cocoa–hazelnut spreads						
FC000	4.33 ± 0.82 ^a^	4.50 ± 0.55 ^a^	4.17 ± 0.41 ^a^	3.67 ± 0.82 ^a^	4.00 ± 0.63 ^a^	4.33 ± 0.52 ^a^
FC025	4.17 ± 0.75 ^a^	4.33 ± 0.52 ^a^	3.83 ± 0.41 ^ab^	3.50 ± 0.55 ^a^	4.00 ± 0.63 ^a^	4.33 ± 0.52 ^a^
FC050	4.33 ± 0.52 ^a^	4.33 ± 0.52 ^a^	4.17 ± 0.41 ^a^	3.17 ± 0.41 ^ab^	3.50 ± 0.55 ^ab^	3.67 ± 0.52 ^ab^
FC075	3.67 ± 0.82 ^ab^	4.00 ± 0.89 ^ab^	3.67 ± 0.52 ^ab^	3.00 ± 0.63 ^ab^	3.33 ± 0.82 ^ab^	3.83 ± 0.41 ^ab^
FC100	3.00 ± 1.10 ^b^	3.67 ± 0.82 ^b^	3.00 ± 0.00 ^b^	2.17 ± 0.75 ^b^	2.67 ± 1.03 ^b^	2.50 ± 0.55 ^b^
Isomaltooligosaccharide-substituted cocoa–hazelnut spreads						
IC000	4.83 ± 0.41 ^a^	4.67 ± 0.52 ^a^	4.33 ± 0.82 ^a^	3.67 ± 0.82 ^a^	4.50 ± 0.55 ^a^	4.50 ± 0.55 ^a^
IC025	4.50 ± 0.55 ^a^	4.50 ± 0.55 ^a^	4.17 ± 0.75 ^a^	3.50 ± 0.55 ^a^	3.50 ± 0.55 ^ab^	4.50 ± 0.55 ^a^
IC050	4.50 ± 0.55 ^a^	4.17 ± 0.98 ^ab^	4.00 ± 0.63 ^a^	3.33 ± 0.52 ^ab^	3.67 ± 0.52 ^ab^	3.83 ± 0.75 ^ab^
IC075	4.00 ± 0.89 ^ab^	4.00 ± 1.26 ^ab^	3.83 ± 0.75 ^ab^	3.00 ± 0.63 ^ab^	3.17 ± 0.75 ^b^	3.17 ± 0.98 ^b^
IC100	2.83 ± 0.98 ^b^	3.00 ± 1.26 ^b^	3.00 ± 0.00 ^b^	2.00 ± 0.63 ^b^	2.50 ± 0.55 ^b^	2.00 ± 0.63 ^c^

Different lowercase superscripts indicate significant differences between sample groups (*p* < 0.05). All experiments were performed in triplicate. All data are represented as means ± standard deviation. FC000–FC100: Cocoa–hazelnut spreads containing 0–100% fructooligosaccharide (FOS) as sucrose replacer. IC000–IC100: Cocoa–hazelnut spreads containing 0–100% isomaltooligosaccharide (IMO) as sucrose replacer.

**Table 5:** Estimated glycemic index of fructooligosaccharide- and isomaltooligosaccharide-substituted cocoa–hazelnut spreads.

Sample	eGI	HI
Fructooligosaccharide-substituted cocoa–hazelnut spreads		
FC000	50.71 ± 0.38 ^a^	20.05 ± 0.70 ^a^
FC025	46.91 ± 0.09 ^b^	13.12 ± 0.16 ^b^
FC050	40.41 ± 0.49 ^c^	1.93 ± 0.05 ^c^
FC075	40.16 ± 0.03 ^c^	0.83 ± 0.05 ^d^
FC100	39.90 ± 0.06 ^c^	0.35 ± 0.10 ^e^
Isomaltooligosaccharide-substituted cocoa–hazelnut spreads		
IC000	51.39 ± 0.16 ^a^	21.28 ± 0.23 ^a^
IC025	48.04 ± 0.13 ^b^	15.18 ± 0.24 ^b^
IC050	43.68 ± 0.14 ^c^	7.24 ± 0.26 ^c^
IC075	42.93 ± 0.05 ^d^	5.86 ± 0.10 ^d^
IC100	41.94 ± 0.67 ^e^	4.53 ± 0.57 ^e^

Different lowercase superscripts indicate significant differences between sample groups (*p* < 0.05). All experiments were performed in triplicate. All data are represented as means ± standard deviation. FC000–FC100: Cocoa–hazelnut spreads containing 0–100% fructooligosaccharide (FOS) as sucrose replacer. IC000–IC100: Cocoa–hazelnut spreads containing 0–100% isomaltooligosaccharide (IMO) as sucrose replacer. eGI: Estimated Glycemic Index. HI: Hydrolysis Index.

## Data Availability

The data supporting the findings of this study are available from the corresponding author upon reasonable request.

## Abbreviations

The following abbreviations are used in this manuscript:

FOS	Fructooligosaccharide
IMO	Isomaltooligosaccharide
eGI	Estimated glycemic index
HI	Hydrolysis index
G′	Storage modulus
G″	Loss modulus
LVR	Linear viscoelastic region
D90	Particle diameter below which 90% of particles are found
PGPR	Polyglycerol polyricinoleate
GOPOD	Glucose oxidase–peroxidase
ANOVA	Analysis of variance
SPSS	Statistical Package for the Social Sciences
L*	Lightness
a*	Redness/greenness coordinate
b*	Yellowness/blueness coordinate
C*	Chroma
h°	Hue angle
WI	Whiteness index
